# Draft Genome Sequence of *Salmonella enterica* subsp. *enterica* Serovar Manhattan Strain 111113, from an Outbreak of Human Infections in Northern Italy

**DOI:** 10.1128/genomeA.00632-13

**Published:** 2013-08-15

**Authors:** Davide Sassera, Stefano Gaiarsa, Erika Scaltriti, Marina Morganti, Claudio Bandi, Gabriele Casadei, Stefano Pongolini

**Affiliations:** Dipartimento di Scienze Veterinarie e Sanità Pubblica (DIVET), Università degli Studi di Milano, Milan, Italya; Istituto Zooprofilattico Sperimentale della Lombardia e dell’Emilia Romagna (IZSLER), Sezione Diagnostica di Parma, Parma, Italyb; Centro di Referenza Nazionale per i Rischi Emergenti in Sicurezza Alimentare, Milan, Italyc

## Abstract

We announce the draft genome sequence of *Salmonella enterica* subsp. *enterica* serovar Manhattan strain 111113, isolated from a patient during an outbreak in northern Italy. The genome, which was obtained with Illumina MiSeq technology, is composed of 21 contigs for a total of 4,684,342 bp, with a G+C content of 52.17%.

## GENOME ANNOUNCEMENT

*Salmonella* infections represent a major food-borne threat, with an estimated 93.8 million cases of gastroenteritis occurring globally each year, resulting in 155,000 deaths (1). *Salmonella enterica* subsp. *enterica* serovar Manhattan is a rare serotype belonging to serogroup C. Only a handful of human outbreaks of *S.* Manhattan have been reported (2–4) worldwide, all caused by contaminated pork-meat products. From May to July 2009, an unusually high incidence of human salmonellosis caused by *S.* Manhattan was registered in a relatively small area in the province of Modena, Italy. Standard and molecular epidemiology investigations revealed the same pulsed-field gel electrophoresis (PFGE) profile for all outbreak-related isolates, all of which were caused by the consumption of pork-meat sausages from a single producer. Here, we announce the availability of the draft genome sequence of one clinical isolate, which will provide unique insights into and a better understanding of this rare serotype.

Whole DNA was extracted using the Qiagen DNeasy blood and tissue kit and was subjected to quality controls. Sequencing was performed on the Illumina MiSeq platform with a 2 × 250 paired-end run, after library preparation with the Nextera XT sample preparation kit (Illumina); 4,231,660 paired sequences were generated, for a total of >1.4 gigabases and a mean length of 171 bases per read. Reads were analyzed and quality checked using FastQC (5) and a specifically designed python script. Genome assembly was performed using Mira 3.4 (6), which resulted in 35 large contigs and an average coverage of 194.52×, for a total of 4,696,349 bp. Genome finishing was performed by aligning the contigs to the closely related genome of *S. enterica* subsp. *enterica* serovar Newport strain SL254 using the software Mauve (7) and by manually checking potential joins using the Gap4 software of the Staden package (8). The finishing process produced a high-quality draft assembly of the strain 111113 genome, consisting of 21 contigs, with a G+C content of 52.17%, for a total of 4,684,342 bp. Genome annotation was automatically performed on the RAST server (9) using Glimmer for base calling, obtaining 4,606 protein-coding genes. The 21 contigs were screened by BLAST analysis against a database containing 24 published *Salmonella* plasmid genomes. No significant hits were retrieved, suggesting the absence of plasmids in the sequenced isolate as confirmed by standard miniprep extraction and agarose gel visualization. Due to the paucity of outbreaks on a worldwide scale, the evolution and pathogenicity characteristics of *S.* Manhattan remain elusive. The availability of this draft genome sequence will enable more-in-depth studies of the epidemiology and virulence mechanisms of this pathogen. A detailed report of a comparative analysis of the whole outbreak will be released in future publications.

### Nucleotide sequence accession numbers.

The genome sequence of *S. enterica* subsp. *enterica* serovar Manhattan 111113 was deposited at EBI under the accession no. CBKW010000001 to CBKW010000021.
